# Using umbilical cord tissue to identify prenatal ethanol exposure and co-exposure to other commonly misused substances

**DOI:** 10.1038/s41372-024-02075-2

**Published:** 2024-07-28

**Authors:** Joseph Jones, Donna Coy, Dominique Gidron, Shanthi Hariharan, Mary Jones, Niranjan Patel, Amy Racines, Sarah Toma, Guida Brown

**Affiliations:** 1 United States Drug Testing Laboratories, Des Plaines, IL USA; 2 Guided by Guida, Kenosha, WI USA

**Keywords:** Diagnostic markers, Epidemiology

## Abstract

**Objective:**

Substance misuse during pregnancy can result in a variety of poor pregnancy outcomes. Objective data reporting the prevalence of neonates born with ethanol metabolites (evidence of prenatal ethanol exposure) in their fluids or tissues are limited.

**Study design:**

A secondary analysis of umbilical cord tissue specimens received for routine toxicological analysis was conducted. Prevalences of ethyl glucuronide (EtG), a long-term direct ethanol biomarker, were determined using a new laboratory tool, LDTD-MSMS. Additionally, other commonly misused substances were determined using routine procedures.

**Results:**

Of 12,995 specimens, 238 (1.8%) specimens contained EtG. Concentrations of EtG ranged from 5 ng/g to 6679 ng/g (median 47 ng/mg; IQR: 16 ng/g, 203 ng/g). Of those 238 EtG-positive specimens, nearly 58% (*N* = 138) contained additional substances or metabolites.

**Conclusion:**

Self-report of substance use during pregnancy is under-reported. We have demonstrated co-exposure of substances with ethanol is higher than previous reports.

## Introduction

Substance misuse during pregnancy can result in poor pregnancy outcomes, including miscarriage, preterm birth, low birthweight, birth defects, and developmental delays [1, 2]. The most common substances used during pregnancy are tobacco, ethanol, and cannabis, with ethanol being the substance most associated with facial dysmorphisms and alterations to central nervous system development [3–5]. Objective data reporting the prevalence of neonates born with ethanol metabolites (evidence of prenatal ethanol exposure) in their system is limited. Ethanol is the most widely misused substance nationally [6], bringing to light the problem of the lack of testing of neonates for ethanol exposure.

A study by England et al. [7] indicated that 10% of pregnant women surveyed in 2015–2018 self-reported current ethanol use, and, of them, 40% also self-reported current use of one or more other substances. In 2022 the Centers for Disease Control and Prevention reported that, from 2018–2020, nearly 14% of pregnant adults in the United States reported current drinking [8, 9]. These self-reports have been determined to underestimate the exact prevalence of substance use in both research and primary healthcare settings, especially when there is a higher social stigma [10], such as substance use during pregnancy.

Biological specimen types available for newborn testing are urine, meconium, blood, or umbilical cord blood or tissue. Umbilical cord tissue as a specimen type is readily available, easily gathered, and easily stored. Umbilical cord tissue has a long window (3rd trimester) for the detection of substances and their metabolites [11–13]. Further, universal collection of umbilical cord tissue allows for unbiased assessment of drug exposure in neonates and provides a medical record to facilitate appropriate diagnoses and therapeutic interventions in the future [14].

Ethyl glucuronide (EtG), a direct long-term ethanol biomarker, is formed by the combination of ethanol and glucuronic acid in the liver, only produced when ethanol is present, and retained in umbilical cord tissue for an undetermined period of time [15]. Detection of EtG in umbilical cord tissue provides objective information for at-risk newborns who were prenatally exposed to ethanol.

The aim of this manuscript is threefold. We will report the prevalence of the detection of EtG in a sampling of umbilical cord tissue specimens received by a commercial reference laboratory. We will report here for the first time the use of a rapid laser diode thermal desorption tandem mass spectrometer (LDTD-MS/MS) with a second aliquot of presumptive positive specimens confirmed using a liquid chromatography tandem mass spectrometer (LC-MS/MS). Lastly, we will report patterns of co-exposure to ethanol and other substances detected in umbilical cord tissue specimens submitted for routine forensic toxicological analysis.

## Experimental

### Materials

Stock standards of 1 mg/mL ethyl-β-D-glucuronide and ethyl-β-D-glucuronide-*d*_*5*_ were purchased from Cerilliant (Round Rock, TX). Certified negative umbilical cord tissue was pooled from previously analyzed specimens sent to the laboratory for routine forensic toxicological analysis and were determined to be negative for EtG. College of American Pathologists Type II water was deionized and filtered using the EVOQUA system (Broadview, IL). All solvents (HPLC grade) were purchased from Thermo-Fisher (Hanover Park, IL USA).

## Methods

### Initial test for EtG in umbilical cord tissue

Specimens were screened for EtG using a LDTD-MS/MS. Aliquots (0.5 g) were homogenized with 3 mL deionized water and 3 stainless-steel wood screws using a Bullet Blender® (Next Advance, Troy, NY, USA) at setting 7 for 5 min. The aliquots were centrifuged at 2000 RPM for 5 min, and the supernatants were loaded onto a Clean-up Quaternary Amine extraction cartridge (United Chemical Technologies, Bristol, PA, USA) that was previously conditioned with 3 mL methanol and 3 mL of deionized water. Extraction cartridges were rinsed with 3 mL acetonitrile followed by 3 mL methanol and eluted with 3 mL 2% formic acid in methanol. After drying at 40 °C, the residues were reconstituted in 200 µL 85:14:1 methanol: deionized water: 0.1 M hydrochloric acid. The extracts were spotted (8 µL) onto AD 96 LazWell™ Plate (Phytronix, Quebec, Canada), dried at 35 °C in a recirculating oven, and analyzed by LDTD-MS/MS. LDTD run time was 9.2 s with a gas flow of 6.0 L/min. Transitions monitored were 226 m/z > 75 m/z for EtG-*d*_*5*_ and 221 m/z > 75 m/z for EtG. Selected mass spectrometer parameters are listed in Table 1.

**Table 1:** Selected LDTD/MS/MS parameters for the initial test of EtG in umbilical cord tissue.

Analyte	Mass Transition	DP (V)	CE(V)	CXP(V)
EtG-*d*_5_	226.0 > 75.0	−100.0	−20.0	−10.0
EtG	221.0 > 75.0	−100.0	−20.0	−10.0

*DP* Declustering Potential, *CE* Collision Energy, *CXP* Collision Cell Exit Potential

### LC-MS/MS procedure for EtG in umbilical cord tissue

The specimens were analyzed using a previously published method [16]. Briefly, specimens were homogenized in 3 mL of acetonitrile, centrifuged, and extracted using the previously referenced anion exchange solid phase extraction method. Eluants were evaporated and reconstituted for analytical analysis. Separation was achieved using an Agilent Technology 1200 HPLC system (Wilmington, DE, USA) fitted with a Synergy Polar RP (50 mm × 2.0 mm, 2.5 um particle size) C-18 column (Phenomenex, Torrence, CA, USA). The solvent system was a gradient that consisted of A (deionized water with 0.1% formic acid) and B (acetonitrile with 0.1% formic acid) using a flow rate of 0.100 mL/min. The solvent program held B at 1% until 2.5 min. Solvent B was increased to 100% between 2.5 min and 2.6 min and held until 3.1 min. Solvent B was lowered to 1% between 3.1 min and 3.2 min and held at 1% until 7.7 min.

The detector was a Sciex Triple Quad^TM^ 5500 tandem mass spectrometer using electro-spray ionization (ESI) in negative mode (Foster City, CA, USA). The ion spray voltage was 4200 V, and the source temperature was 650 ˚C. The curtain and collision gases were nitrogen and were held at 30 psi and 5 psi, respectively. The internal standard (EtG-*d*_*5*_) was monitored using the m/z 226.1 > 75.0 (quantification ion; declustering potential [DP] = 65 V; collision energy [CE] = 22 V; collision cell exit potential [CXP] = 13 V) transition; and the m/z 226.1 > 85.0 (qualifying ion; DP = 65 V; CE = 22 V; CXP = 13 V) transition. The m/z 221.1 > 75.0 (quantification ion; DP = 37 V; CE = 21 V; CXP = 22 V) and m/z 221.1 > 85.0 (qualifying ion; DP = 44 V; CE = 23 V; CXP = 22 V) transitions were used to monitor EtG. The dwell time for monitoring each transition was 400 ms.

### Initial testing and confirmation of other substances in umbilical cord tissue

Umbilical cord tissues were analyzed at United States Drug Testing Laboratories (USDTL; Des Plaines, IL, USA) for amphetamines, cocaine, opiates, phencyclidine, cannabinoids, barbiturates, benzodiazepines, propoxyphene, methadone, oxycodone, meperidine, tramadol, fentanyl, buprenorphine, ketamine, gabapentin, and mitragynine. Specimens were initially tested using enzyme linked immunosorbent assays (ELISA). The product vendor and cutoffs are listed in Table 2. Presumptive positive specimens were confirmed using LC-MS/MS or gas chromatography-tandem mass spectrometry. Selected confirmation performance characteristics are listed in Table 3.

**Table 2:** Reagent manufacturers and cutoffs for immunoassay initial tests.

Drug Class	Manufacturer	Cutoff (ng/g)
Methamphetamines	Neogen	5
Cannabinoids	Neogen	0.1
Cocaine	Neogen	0.5
Opiates	Immunalysis	0.5
Phencyclidine	Immunalysis	2
Methadone	Immunalysis	2
Barbiturates	Immunalysis	1
Benzodiazepines	Immunalysis	2
Propoxyphene	Neogen	4
Oxycodone	Immunalysis	0.5
Meperidine	Neogen	2
Tramadol	Immunalysis	4
Buprenorphine	Immunalysis	0.5
Cotinine	Immunalysis	5
Fentanyl	Immunalysis	0.2

**Table 3:** Selected performance characteristics of confirmation assays for commonly misused substances.

Analyte	Cutoff (ng/g)	LOD (ng/g)	LOQ (ng/g)	ULOQ (ng/g)
Amphetamine	5.0	1.0	2.0	100
Methamphetamine	5.0	1.0	2.0	100
MDA	5.0	1.0	2.0	100
MDMA	5.0	1.0	2.0	100
MDEA	5.0	1.0	2.0	100
Benzoylecgonine	0.5	0.1	0.2	20
Codeine	0.5	0.1	0.2	20
Morphine	0.5	0.1	0.2	20
Hydrocodone	0.5	0.1	0.2	20
Hydromorphone	0.5	0.1	0.2	20
6-MAM^a^	0.2	0.04	0.08	20
Meconin	0.2	0.04	0.08	20
Phencyclidine	1.0	0.2	0.4	100
Carboxy-THC	0.05	0.01	0.02	1.0
Butalbital	1.0	0.2	0.4	20
Amobarbital	1.0	0.2	0.4	20
Pentobarbital	1.0	0.2	0.4	20
Secobarbital	1.0	0.2	0.4	20
Phenobarbital	1.0	0.2	0.4	20
Diazepam	2.0	0.4	0.8	40
Nordiazepam	2.0	0.4	0.8	40
Oxazepam	2.0	0.4	0.8	40
Temazepam	2.0	0.4	0.8	40
Alprazolam	2.0	0.4	0.8	40
Midazolam	2.0	0.4	0.8	40
Methadone	2.0	0.4	0.8	40
EDDP^b^	2.0	0.4	0.8	40
Propoxyphene	2.0	0.4	0.8	40
Norpropoxyphene	2.0	0.4	0.8	40
Oxycodone	0.5	0.1	0.2	20
Oxymorphone	0.5	0.1	0.2	20
Tramadol	2.0	0.4	0.8	40
Meperidine	2.0	0.4	0.8	40
Normeperidine	2.0	0.4	0.8	40
Buprenorphine	0.25	0.05	0.1	12.5
Norbuprenorphine	1.0	0.2	0.4	50
Fentanyl	0.2	0.08	0.04	4
Norfentanyl	0.2	0.08	0.04	4
Acetyl fentanyl	0.2	0.08	0.04	4
Acetyl norfentanyl	0.2	0.08	0.04	4
Gabapentin	10.0	2.0	4.0	100
Mitragynine	5.0	1.0	2.0	100
Cotinine	5.0	1.0	2.0	400

*LOD* Limit of Detection, *LOQ* Limit of Quantitation, *ULOQ* Upper Limit of Quantitation, ^a^6MAM = 6-Monoacetylmorphine; ^b^EDDP = 2-Ethylidene-1,5-dimethyl-3,3-diphenylpyrrolidine.

### Specimens

This secondary analysis utilized de-identified historical data for umbilical cord tissue specimens received for routine toxicological analysis at a national reference laboratory (United States Drug Testing Laboratories, Des Plaines, IL, USA). Umbilical cord tissue selected for analysis fit local hospital criteria and were collected following established procedures (https://www.usdtl.com/). Specimens were refrigerated following collection and shipped overnight at ambient temperature to the laboratory using a commercial courier. Following receipt at the laboratory, testing was initiated the same day and stored refrigerated until all testing was complete. Institutional review board approval was not required for secondary analysis of de-identified results. As per guidance in 45CFR 46.104(d)4(ii), our study qualified as IRB Exempt Research because we studied de-identified biological specimens with no access to human subject information, and because the identity of the human subjects could not readily be ascertained directly or through identifiers linked to the subjects [17]. Prevalence, medians, and interquartile ranges were calculated using Excel. Additionally, co-exposure use patterns were evaluated.

## Results

During the study period, the laboratory received 12,995 umbilical cord tissue that were analyzed for EtG. Two-hundred and thirty-eight (1.8%) specimens were positive for EtG ( >5 ng/g). The concentrations for EtG ranged from 5 ng/g to 6679 ng/g (median 47 ng/mg; IQR: 16 ng/g, 203 ng/g).

Of those 238 EtG-positive specimens, nearly 58% (*N* = 138) were reported positive for an additional substance or metabolite. There were 100 specimens positive for only EtG (42%), whereas the number of specimens with 1 additional exposure was 88 (36.9%), 2 additional exposures was 38 (15.9%), 3 additional exposures was 10 (4%), and 4 additional exposures was 2 (1%). Among the multiple positive specimens, carboxy-Δ-9-THC was the most frequent co-exposure (*N* = 63; 26.5%). Amphetamines were the second most frequent co-exposure with 55 (23.1%) followed by cocaine (*N* = 23; 9.6%), opiates (*N* = 18; 7.5%), cotinine (*N* = 11; 4.6%), buprenorphine (*N* = 9; 3.7%), fentanyl (*N* = 7; 2.9%), benzodiazepine (*N* = 5; 2.1%), barbiturate (*N* = 3; 1.2%), gabapentin (*N* = 3; 1.2%), methadone (*N* = 2; 0.8%), mitragynine (*N* = 2; 0.8%) and tramadol (*N* = 1; 0.4%).

## Discussion

We reported here for the first time a high throughput LDTD-MS/MS initial test for a long-term ethanol biomarker, EtG, in umbilical cord tissue coupled with a reflex of presumptive positive specimens to a definitive LC-MS/MS confirmatory method for the purpose of objectively identifying prenatal ethanol exposure. During the study period, there was sufficient umbilical cord tissue to complete all requested analyses on all specimens received at the laboratory, which is a significant improvement over other newborn specimen types such as neonatal urine, meconium, and hair where 10–28% of the cases (depending on the specimen type and circumstances) were reported unavailable for testing due to missed collection or quantity not sufficient to complete testing [12, 13, 18, 19]. Additionally, the analysis time was improved with the transition from an LC-MS/MS initial test (run time approximately 9 min) to a LDTD-MS/MS initial test (run time approximately 9 s), allowing for up to a 60-fold increase of reportable results per instrument per equivalent time period.

Our study found that 1.8% of the umbilical cord tissue received for testing were positive for EtG, which implies that 1.8% of this demographic were exposed to ethanol in utero. While this was a simple convenience sampling and not a thoroughly planned epidemiological study, a 1.8% positivity rate is concerning and requires further inquiry. England et al. [7] reported that 10% of the pregnant persons participating in their survey had self-reported current ethanol use, which included pregnant persons at all stages of fetal development and not necessarily near the time of delivery. Ethen et al. [20], reporting their analysis of the National Birth Defect Prevention Study, noted that 7.4% of mothers who recently delivered a healthy child without an apparent birth defect self-reported consuming ethanol during the third trimester and that 0.5% reported binge drinking during the third trimester. More research is required to better understand the formation, elimination, and the detection time window of EtG in umbilical cord tissue.

We reported that 58% of our cases contained at least one other substance in addition to the ethanol biomarker, EtG. Our co-exposure finding of 58% exceeds a previous study [7] that reported that 40% of the mothers who self-reported consuming ethanol during pregnancy also consumed other substances. This comparison is an additional piece of evidence of the importance of the complementary nature of self-report and biomarker testing. The potential negative consequences of prenatal exposure to ethanol have been well-established in the Fetal Alcohol Spectrum Disorders (FASD) literature [21, 22, 23]; however, studies investigating the potential impact of co-exposure to other substances with ethanol are limited.

There are several limitations that restrict the generalizability of our study that should be noted. This study utilized a convenience sampling of specimens submitted to our laboratory for analysis. The selection criteria of the cases submitted to our laboratory for testing were blind to the laboratory. This study did not have access to the medical records for the cases presented here and therefore did not include a birth outcome analysis. The specimens were only tested for substances requested by the hospital; therefore, the co-exposure reported here may be underestimated. We were not able to determine if the co-exposures occurred simultaneously or over different periods of time within the detection window. Although the detection window for substances in umbilical cord tissue has not been definitively determined, it is generally thought to include only the third trimester, which means that substance use activity in the periconceptual period and early trimesters are not detected [24].

## Conclusion

Although self-report of substance use during pregnancy provides vital information for pregnancy outcomes, actual prevalence of substance use is likely under-reported. In this study we report the prevalence of EtG in umbilical cord tissue specimens received at our laboratory. Timely reporting of toxicology results is key in neonatal testing. Utilization of LDTD-MS/MS as an initial screening tool to rapidly detect EtG in 9 s, resulting in a 60-fold increase in efficiency of reporting. In addition, we have demonstrated that co-exposure of substances with ethanol is significant. More studies are needed to fully understand the prevalence and implications of EtG detection in umbilical cord tissue as an indicator of ethanol exposure and co-exposure to other substances during pregnancy.

## Data Availability

The data may be made available from the corresponding author on reasonable request.
